# Why do He and She Disagree: The Role of Binary Morphological Features in Grammatical Gender Agreement in German

**DOI:** 10.1007/s10936-022-09926-z

**Published:** 2023-01-16

**Authors:** Margret Seyboth, Frank Domahs

**Affiliations:** Department of Linguistics, University of Erfurt, P. O. Box 90 02 21, 99105 Erfurt, Germany

**Keywords:** Grammatical gender, Binary features, Underspecification, Markedness, Inflection

## Abstract

In many languages, grammatical gender is an inherent property of nouns and, as such, forms a basis for agreement relations between nouns and their dependent elements (e.g., adjectives, determiners). Mental gender representation is traditionally assumed to be categorial, with categorial gender nodes corresponding to the given gender specifications in a certain language (e.g., [masculine], [feminine], [neuter] in German). In alternative models, inspired by accounts put forward in theoretical linguistics, it has been argued that mental gender representations consist of sets of binary features which might be fully specified (e.g., masc [+ m, − f], fem [− m, + f], neut [− m, − f]) or underspecified (e.g., masc [+ m], fem [+ f], neut [] or masc [+ m, − f], fem [], neut [− f]). We have conducted two experiments to test these controversial accounts. Native speakers of German were asked to decide on the (un-)grammaticality of gender agreement of visually presented combinations of I) definite determiners and nouns, and II) anaphoric personal pronouns and nouns in an implicit nominative singular setting. Overall, agreement violations with neuter *das* / *es* increased processing costs compared to violations with *die / sie* or *der / er* for masculine or feminine target nouns, respectively. The observed pattern poses a challenge for models involving categorial gender representation. Rather, it is consistent with feature-based representations of grammatical gender in the mental lexicon.

## Introduction

Grammatical gender is an “inherent property of nouns which controls morphologically marked agreement relations between different syntactic elements” (Bußmann, 2002, p. 247, transl.). Thus, “at least one other part of speech (determiner, adjective, pronoun) carries corresponding morphological features” (Bußmann, 2002, p. 247, transl.). The existence of grammatical gender as well as its function and formal marking and the number of gender specifications vary between languages (e.g., Corbett, 2014).

German differentiates between three gender specifications—*masculine*, *feminine,* and *neuter*. In nominative singular, the definite determiners *der*, *die*, and *das* and the anaphoric personal pronouns *er*, *sie*, and *es* are associated with these gender specifications. Table 1 displays the inflection paradigm of the German definite determiners and anaphoric personal pronouns.

**Table 1:** Inflection paradigm of the German definite determiners and anaphoric personal pronouns

	Singular			Plural
	m	f	n	m / f / n
Nominative	der / er	die / sie	das / es	die / sie
Genitive	des / seiner	der / ihrer	des / seiner	der / ihrer
Dative	dem / ihm	der / ihr	dem / ihm	den / ihnen
Accusative	den / ihn	die / sie	das / es	die / sie

As can be deduced from Table 1, morphological markers within the inflection paradigm differ as a function of gender specification. This may apply to the nouns themselves as well as to dependent parts of speech.

### Representation of Grammatical Gender in Psycholinguistic Models

**Fig. 1:**
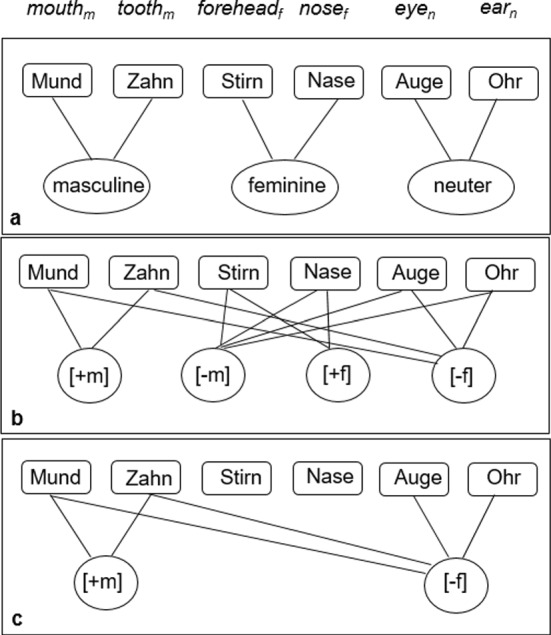
Gender representation within the discrete two step speech production model **a** with categorial gender representation (e.g., Jescheniak & Levelt, 1994), **b** with decomposed gender representation (e.g., Penke et al., 2004; Opitz et al., 2013), and **c** with decomposed and underspecified gender representation (Opitz & Pechmann, 2016; model adapted from Jescheniak & Levelt, 1994, p. 826 and Opitz & Pechmann, 2016, p. 236)

According to psycholinguistic models like the discrete two step speech production model of Levelt and colleagues (e.g., Jescheniak & Levelt, 1994; Levelt, 1989, 1999, 2001), gender information in the mental lexicon is stored at a modality independent lemma level. There, each gender specification is represented by one central gender node, which is connected to all nouns of this gender specification (e.g., Jescheniak & Levelt, 1994; cf. Figure 1a). The number of gender nodes equals the number of gender types in a given language, that is, for German, three gender nodes are assumed.

As the classical discrete two step model implies that gender representations are equivalent to the three gender specifications, speed of gender access and processing should not differ for masculine, feminine, and neuter words, if other semantic, lexical, and morphosyntactic features are held comparable across these specifications.

There is, however, a number of studies suggesting that nouns within the mental lexicon are not connected to abstract gender nodes, but to feature nodes representing gender in a decomposed way as illustrated in Fig. 1b.

This assumption is based on theoretical linguistic frameworks like Distributed Morphology (e.g., Halle & Marantz, 1994) or Minimalist Morphology (e.g., Wunderlich, 1996) which propose that morphosyntactic specifications are composed of abstract binary features which have either a positive (marked) or a negative (unmarked) value. In such accounts, German gender specifications are supposed to be realised via the features masculine [+ / − m] and feminine [+ / − f]. Specifically, masculine and feminine gender specifications are marked in a complementary way, while neuter forms are unmarked, i.e., masculine = [+ m, − f], feminine = [− m, + f], and neuter = [− m, − f] (e.g., Bierwisch, 1967, p. 248). Other morphosyntactic categories, too, can be decomposed in this way. Thus, grammatical number can be specified as singular = [-pl] and plural = [+ pl] (e.g., Bierwisch, 1967, p. 248) and case as nominative = [− obj(ect), − obl(igatory)], accusative = [+ obj, − obl], dative = [+ obj, + obl], and genitive = [− obj, + obl] (e.g., Opitz et al., 2013, p. 235). 

According to morphological underspecification accounts, grammatical elements may not necessarily be fully specified for morphological properties, but may lack features, even though every incidence of this element in a syntactic context is specified for these features (Lehmann, n.d.). This might result in an underspecified feature representation within the mental lexicon as has been suggested by Opitz and Pechmann (2016; cf. Figure 1c).

### Empirical Evidence for Decomposed Gender Representation in the Mental Lexicon

First evidence for decomposed gender representation in the mental lexicon was put forward by Clahsen et al. (2001) who conducted a visual lexical decision experiment involving inflected adjectives. They found that processing of adjectives with a very specific affix (*− m* with two positive features: [+ obj, + obl]) resulted in longer reaction times (RTs) compared to processing of adjectives with a less specific affix (− *s* with no positive feature). Furthermore, they conducted a cross-modal priming experiment with auditory primes and visual stimuli. There, priming effects were smaller for specific affixes whose morphosyntactic features were primed incompletely compared to less specific affixes whose morphosyntactic features were fully primed. However, as Opitz et al. (2013) emphasise, it cannot be ruled out that the results were confounded by phonological features. As they argue, e.g., the affix *-e* was fully primed by the affix − *(e)s*, but this was not the case in the opposite condition (stimulus: − *(e)s*, prime: − *e*).

Janssen and Penke (2002) analysed errors of person and number agreement between subjects and verbs in sentence completion and elicitation tasks with German agrammatic patients. They found, amongst others, that in most cases, marked features were replaced by unmarked features.

Penke et al. (2004) compared reading times for correct and incorrect sentences in a sentence-matching test. Sentences included prepositional adjective (Experiment I) and determiner (Experiment II) noun phrases with matching or non-matching inflectional markers. Typically, in such a test, incorrect sentences induce longer RTs than correct sentences. This grammaticality effect, however, emerged only in sentences where positive features of an inflected form were missing or negatively specified in the syntactic context. It did not emerge when negative or missing features of an inflected form co-occurred with positively specified features in the syntactic context. Penke and colleagues interpret these results as indication of a relevant distinction between the two principles of compatibility and specificity. That is, an underspecified morphosyntactic element is chosen for a given context when it is (a) compatible with this context and (b) the most specific of all elements fulfilling precondition (a) (e.g., Opitz et al., 2013). Violations of compatibility are supposed to be more serious than violations of specificity and, thus, may result in different processing costs. Furthermore, Penke and colleagues argue that positive features are part of the representation of morphologically complex words or affixes while negative features are applied on the basis of the paradigmatic context. Therefore, only positive features can disagree with the syntactic context and, thus, decelerate RTs. Results, however, are to be interpreted with caution since in some sentences the mismatch of the inflectional markers became apparent already with the combination of preposition and adjective while in others it turned up first with the combination of adjective and noun. This might explain some RT-differences irrespective of the occurrence of positively marked features in the context.

Opitz et al. (2013) asked German subjects to rate the grammaticality of visually presented prepositional accusative adjective-noun phrases. Each noun was combined with each adjective three times, containing a masculine, a feminine, or a neuter accusative marker, respectively. Overall, phrases with masculine nouns were more error-prone than phrases with feminine or neuter nouns. Additionally, event related potentials (ERPs) were analysed at the time of noun presentation. In all incorrect conditions, a P600 occurred, i.e., a positive deflection at 600–900 ms after presentation of the critical stimulus. The P600 is associated with syntactic processing difficulties or the need of a reanalysis (e.g., Frisch et al., 2002; Gouvea et al., 2009). Within the experiment, it indicated different processing of correct and incorrect phrases. Additionally, a left anterior negativity (LAN) was observed 300–550 ms after presentation of the noun. It is associated with the identification of a morphosyntactic error. In phrases with masculine and feminine nouns, there were no LAN-differences between the two incorrect conditions. Furthermore, in phrases with masculine nouns, there was no difference between incorrect and correct conditions. However, in phrases with neuter nouns, the LAN was larger in combinations with masculine adjectives—corresponding to a violation of compatibility—compared to feminine adjectives—corresponding to a violation of specificity. The authors interpret their results in terms of a feature-based account assuming maximal underspecification of features and a generally increased processing effort for masculine nouns (for a suggested explanation see the next paragraph).

The study of Opitz et al. (2013) was complemented by an experimental series of Opitz and Pechmann (2016). Experiment I was a replication of the experiment described above, but this time RTs served as dependent variable. In Experiment IIa, participants decided whether visually presented nouns were masculine or feminine. In Experiment IIb, participants decided whether visually presented nouns conformed to the gender specification of a given block of words. In Experiment III, participants decided whether a visually presented word was a noun or not; verbs and adjectives served as fillers. Overall, masculine nouns induced more errors and longer RTs compared to feminine nouns across all experiments, while neuter nouns were taking a middle position. According to Opitz and Pechmann (2016), these results reflect differential processing efforts for representatives of the different gender specifications resulting from a different number of connections to gender feature nodes. Processing effort would be directly related to the number of gender features to be activated and retrieved. Opitz and Pechmann state that their experimental results speak in favour of least specified feminine nouns and most specified masculine nouns. Thus, feminine nouns would be the default gender specification with no connection to any feature node; neuter nouns would be connected to one gender feature ([− f]), and masculine nouns to two features ([− f] and [+ m]; see Fig. 1c).

All in all, Opitz and Pechmann question not only a categorial gender representation in favour of feature-based representations but also the restriction of underspecified gender representations to the domain of inflectional markers. Instead, they suggest that underspecification “is more broadly used in the mental lexicon and extends to the feature specification of nouns” (Opitz & Pechmann, 2016, p. 235). While it is conceivable that gender representation in the mental lexicon is based on features, the claim that underspecification of gender extends to nouns can, in our view, be called into question for empirical as well as theoretical reasons:

First, Opitz and Pechmann’s (2016) assumption of underspecified nouns relies heavily on the observation of longer RTs for masculine compared to feminine nouns in a series of experiments. However, this observation may be an artefact of confounding variables of the specific stimuli chosen. Even though nouns were controlled for frequency and length and phrases for plausibility and familiarity, the stimulus words differed critically regarding several formal characteristics. Particularly, 33 of 60 feminine nouns had a schwa-ending strongly associated with feminine gender (e.g., Wegener, 1995). No such clear formal cues existed for the masculine or the neuter nouns. In addition, due to their productivity and frequency, feminine morphological gender indicators were potentially easier to recognise within the experiments than masculine and neuter morphological markers of the nouns. Furthermore, 51 of 60 feminine nouns, but only 29 of 60 masculine and 28 of 60 neuter nouns had their stress on the first syllable which is the prototypical stress pattern of German nouns and, therefore, might also be associated with shorter phonological processing times (e.g., Sulpizio et al., 2015). Taken together, confounding variables such as cue validity or word stress—or others—might account for the experimental results (i.e., the masculine disadvantage) regarded as critical by Opitz and Pechmann (2016) without drawing on underspecified feature-based gender representations of nouns in the mental lexicon.

Second, for theoretical reasons it seems as if the assumption of underspecification of gender features in both the context *and* the dependent words undermines the whole idea of decomposition and underspecification. Underspecification accommodates for the fact that the same morphological markers appear in different syntactic contexts. For example, the German determiner *dem* is only specified for [− f] and can, thus, be applied to masculine or neuter contexts which are thought to be characterised as [+ m, − f] and [− m, − f], respectively. With underspecification of both the dependent word *and* the noun, not only the dependent word but also the noun can be combined with any other dependent word whose specification does not contradict its own specification. In consequence, the mapping direction seems to become somewhat arbitrary. Thus, if a noun like *Klima*_neut_ “climate” was only specified as [− f], it should be possible to combine this noun not only with other neuter [− f] but also with masculine [+ m, − f] depending words neither of which disagrees with the [− f] specification of *Klima*. This is clearly not the case. For example, the incongruent combination *der*_masc_ *Klima*_neut_ would be identified as erroneous by a contextual specification including a fully specified noun (*der_masc_ [+ m, − f] *Klima*_neut_ [− m, − f]) but would be accepted under the assumption of underspecification of both determiner *and* noun (*der_masc_ [+ m, − f] *Klima*_neut_ [− f]).

Thus, empirical as well as theoretical considerations result in scepticism towards the assumption of underspecified feature-based gender representations of nouns. Therefore, this assumption will not be considered anymore hereinafter. Opitz and Pechmann’s (2016) assumption, that the total number of features is relevant for processing costs, however, seems plausible and is taken up in the hypotheses.

### The Present Study

In summary, there are two fundamentally different types of approaches to grammatical gender representation in the mental lexicon. On the one hand, predominant psycholinguistic models postulate categorial gender nodes. On the other hand, recent experimental results suggest that feature decomposition as discussed in theoretical linguistics is also the basis of mental representations and processing of morphological properties, including grammatical gender. Within such decomposition approaches, full specification of features as well as different kinds of underspecification are being discussed. Also, different suggestions have been made regarding the kind of feature processing which leads to specific response patterns in RTs, ERs, or electrophysiological potentials (e.g., relevance of all features involved vs. relevance of positive features of the dependent word only vs. relevance of feature compatibility and specificity).

The present study aimed at testing hypotheses resulting from these different suggestions. To this end, two experiments on gender agreement in visually presented combinations of definite determiners and nouns (Experiment I) as well as anaphoric personal pronouns and nouns (Experiment II) were conducted with German speaking participants. Every noun was combined with each of the three possible definite determiners or personal anaphoric pronouns in nominative case. Error rates (ERs) and reaction times (RTs) were compared for the incorrect combinations of the nouns with their two incongruent determiners or pronouns (agreement violations). Comparisons of ERs and RTs in the (congruent) agreement conditions across the three gender specifications were not considered in detail. This was to accommodate for the fact that different nouns differ not only regarding lexical-semantic characteristics like abstractness, frequency, or length, but also regarding gender-specific factors like availability and reliability of semantic, morphological, or phonological gender indicators (e.g., Köpcke, 1982; see also the General Discussion). It seems virtually impossible to fully parallel nouns across gender specifications for all possible confounding variables.

Based on the explanatory approaches described above, the following hypotheses regarding the comparison of the incongruent conditions could be deduced:

#### Categorial Representation of Grammatical Gender (Classical Discrete Two-Step Model, e.g., Jescheniak & Levelt, 1994)

With the assumption of categorial gender representation, ERs and RTs should be similar across the two possible agreement violation conditions for each gender specification (cf. Table 2). However, differences could potentially arise from (a) differences of frequency of the three gender specifications or word forms of determiners / pronouns, (b) formal differences between the determiners / pronouns (e.g., different number of phonemes or graphemes, different degrees of phonemic / graphemic similarity), or (c) differential compatibility of formal or semantic gender markers of the noun and the incongruent determiner / pronoun. Thus, for example, 64% of the German one-syllable words are masculine, 22% neuter, and 14% feminine. In the light of this distribution, the presentation of a neuter one-syllable word like *Haus* “house” might result in a faster rejection of *die / sie* compared to *der / er* simply because *die / sie* is less frequently associated with a one-syllable noun than *der / er*. Furthermore, even in a nominative context, rejection of feminine nouns with *das* might be faster than rejection of feminine nouns with *der* as *der* can also appear in feminine genitive singular contexts, while *das* is not represented in any cell of the feminine paradigm (cf. Table 1). The personal pronouns *es* and *er*, however, are unique to masculine and neuter nominative singular contexts, respectively, and should therefore produce comparable RTs for combinations with feminine nouns, based on their validity (but ignoring their frequency of use).

**Table 2:** Examples for stimulus combinations (left panel) and predictions for comparison of the two agreement violation conditions per gender specification (right panel) based on the assumption of categorial gender nodes

Noun gender	der / er	die / sie	das / es	ER / RT
Masculine	der / er Mantel,”coat”	*die / *sie Mantel	*das / *es Mantel	die = das / sie = es
Feminine	*der / *er Party,”party “	die / sie Party	*das / *es Party	der = das / er = es
Neuter	*der / *er Klima,”climate “	*die / *sie Klima	das / es Klima	der = die / er = sie

*Denotes agreement violations

*ER* error rate, *RT* response time

#### Feature-Based Representations of Grammatical Gender

Regarding feature-based representations of grammatical gender, different explanation attempts have been made regarding (a) full specification or underspecification, and (b) relevant processing aspects like number of features involved or violation of compatibility vs. specificity. Even (c) the question may be raised of whether the morphosyntactic context is specified serially from left to right (i.e., in chronological order) or by the noun as the syntactic head of a combination. Combinations of these different dimensions result in a considerable number of different possible predictions regarding expected RT patterns in the violation conditions, which cannot be presented here in detail. Based on previous proposals, hypotheses can be derived as follows (cf. Table 3):

**Table 3:** Predictions for comparison of the two agreement violation conditions per gender specification based on the assumptions put forward by Penke et al. (2004), Opitz et al. (2013), and Opitz and Pechmann (2016)

Study	Penke et al. (2004)			Opitz et al. (2013)			Opitz and Pechmann (2016)		
Relevant aspect of processing	Missing positive features in the context			Violation of compatibility (C) versus specificity (S)			Number of features involved		
Assumed feature representation	der / er [+ m]	die / sie [+ f]	das / es []	der / er [+ m, − f]	die / sie []	das / es [− f]	der / er [+ m, − f]	die / sie []	das / es [− f]
nouns_masc_ [+ m, − f]	die / sie (1) < das / es (0)			die / sie (S) = das / es (S)			die / sie (2) < das / es (3)		
nouns_fem_ [− m, + f]	der / er (1) < das / es (0)			der / er (C) = das / es (C)			der/er (4) > das / es (3)		
nouns_neut_ [− m, − f]	der / er (1) = die / sie (1)			der / er (C) < die / sie (S)			der/er (4) > die / sie (2)		

^*^The numbers in parentheses indicate the numbers of critical features, resulting in more or less processing costs

Thus, Penke et al. (2004) postulate underspecified feature representations for the dependent words with masc [+ m], fem [+ f], and neut [], while nouns are fully specified regarding gender (masc [+ m, − f], fem [− m, + f], neut [− m, − f]). Grammaticality effects are only observed when a positive feature of the dependent word is missing or negatively specified in the context. In a task which involves the detection of morphosyntactic violations, thus, in combinations with masculine and feminine nouns, *die / sie* and *der / er* respectively should induce less errors and shorter RTs than *das / es* (e.g., *die* [+ f] *Mantel* [+ m, − f] < *das* [] *Mantel* [+ m, − f]*, der* [+ m] *Party* [− m, + f] < *das* [] *Party* [− m, + f]). Similar RTs are to be expected for *der / er* and *die / sie* in combinations with neuter nouns (e.g.,*der* [+ m] *Klima* [− m, − f] = *die* [+ f] *Klima* [− m, − f]).

According to Opitz et al. (2013, p. 246 and 254), differences in processing costs result from the degree of feature agreement violation between partly underspecified dependent words with masc [+ m, − f], fem [], and neut [− f]) and fully specified nouns (masc [+ m, − f], fem [− m, + f], neut [− m, − f]). Violations of compatibility (e.g., [+ m, − f] − [− m, + f] are easier to detect and, thus, result in less errors and shorter RTs than violations of specificity (e.g., [− f] − [+ m, − f]). Therefore, similar RTs are to be expected for *die / sie* as well as *der / er* and *das / es* in combinations with masculine and feminine nouns respectively (e.g., *die* [] *Mantel* [+ m, − f] = *das* [− f] *Mantel* [+ m, − f] → in both cases violation of specificity*, der* [+ m, − f] *Party* [− m, + f] = *das* [− f] *Party* [− m, + f] → in both cases violation of compatibility). In combinations with neuter nouns, *der / er* should induce less errors and shorter RTs than *die / sie* (e.g., *der* [+ m, − f] *Klima* [− m, − f] → violation of compatibility < *die* [] *Klima* [− m, − f] → violation of specificity).

Finally, Opitz and Pechmann (2016) argue that the absolute number of features involved in processing critically affects RTs. Furthermore, they postulate underspecified feature representations not only for the dependent word but also for the noun. While we do not agree with this latter assumption for the reasons discussed above, the other assumptions seem plausible. Thus, combinations of masculine nouns with *die / sie* would induce less errors and faster RTs than combinations of masculine nouns with *das / es* (e.g., *die* [] *Mantel* [+ m, − f] < *das* [− f] *Mantel* [+ m, − f]). In combinations with feminine as well as neuter nouns, *der/er* would result in more errors and longer RTs compared to *das / es* and *die / sie,* respectively (e.g., *der* [+ m, − f] *Party* [− m, + f] > *das* [− f] *Party* [− m, + f], *der* [+ m, − f] *Klima* [− m, − f] > *die* [] *Klima* [− m, − f]).

Yet, even if none of these accounts may be entirely correct, principally, differences in RTs in the violation conditions speak in favour of some kind of decomposition of gender representation in the mental lexicon.

## Experiments

### Experiment I: Gender Agreement Decision for Determiner Noun Phrases

In order to test the predictions presented above, in a first experiment, ERs and RTs were measured for decisions on gender agreement between definite determiners and nouns.

#### Materials and Procedure

One hundred and twenty morphologically simple German nouns served as stimuli for this experiment, 40 for each gender specification (masculine, feminine, neuter). Word length measured in number of syllables and graphemes as well as type frequency and lemma frequency according to dlex^1^ were matched across gender specifications. Target nouns are listed in Appendix Table 11. As Experiment I was embedded in an experiment on compound processing, 60 compound nouns served as fillers. During the experiment, each noun appeared three times—once with each of the three definite determiners (e.g., *das Kleid* “*dress*”, neut.-*die Kleid-*der Kleid)–in randomised order.

Stimuli were displayed visually in the centre of a computer screen using the DMDX software (http://www.u.arizona.edu/~kforster/dmdx/dmdx.htm; cf. Forster & Forster, 2003). First, a fixation cross appeared for 500 ms. It was followed by the determiner-noun phrase whose parts (determiner and noun) were presented simultaneously, horizontally aligned in left-to-right order (i.e. in the default order of German noun phrases). Participants were instructed to decide as fast and accurately as possible on gender agreement of determiner and noun by pressing the corresponding button (YES or NO). YES answers were assigned to the participant’s dominant hand. Stimulus presentation was terminated by the participant’s response or automatically after 3000 ms. Subsequently, a new trial started automatically.

The experimental testing was preceded by 18 practice trials in order to familiarise participants with the task. Afterwards, all 540 nominal phrases (target nouns and fillers) were presented, with pauses at an interval of 60 trials. Overall, the experiment took 30–40 min. Correctness of the answers and RTs were recorded with DMDX.

#### Participants

Thirty native speakers of German took part in the experiment. With one exception, all of them were students at the University of Erfurt. 23 of them were female, seven male. Mean age was 22.6 years (range: 18–41). None of the participants was diagnosed with dyslexia. Four of them were left-handed. Participants were paid for their participation.

#### Results

Incorrect responses, responses lasting longer than 3000 ms, and responses exceeding 2.5 standard deviations of a participant’s individual RT mean (calculated separately for YES and NO answers) were counted as errors. Across the 360 experimental target stimuli, no participant exceeded an ER of 11% (mean ER: 6.2%, range: 3.3–10.6%).

##### Error Rates

**Fig. 2:**
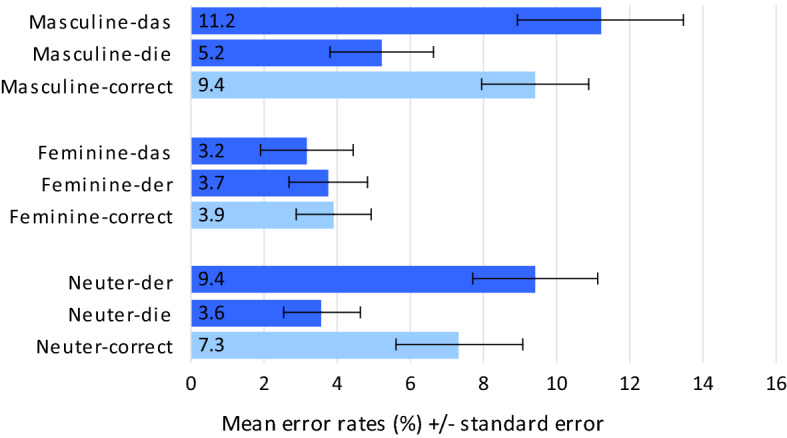
Mean error rates (%) in Experiment I (determiner noun agreement decision)

Results of the ER analyses are summarised in Fig. 2. Most relevant with respect to the different hypotheses are comparisons between the two agreement violation conditions per gender specification.

The ERs in the agreement violation conditions were compared with paired t-tests across participants and items.

*Agreement violation conditions with masculine nouns* Comparison of ERs in masculine nouns revealed a significant difference with more errors on combinations with neuter *das* (mean_masc_das_ = 11.2%, SD = 14.2) compared to combinations with feminine *die* (mean_masc_die_ = 5.2%, SD = 8.9; t_1_(29) = 4.075, *p* < 0.001; t_2_(38) = 3.077, *p* = 0.004).

*Agreement violation conditions with feminine nouns* Comparison of ERs in feminine nouns revealed no significant difference between combinations with neuter *das* (mean_em_das_ = 3.2%, SD = 8.1) compared to combinations with masculine *der* (mean_fem_der_ = 3.7%, SD = 6.8; t_1_(29) = − 0.361, *p* = 0.721; t_2_(39) = − 0.606, *p* = 0.548).

*Agreement violation conditions with neuter nouns* Comparison of ERs in neuter nouns revealed a significant difference with more errors on combinations with masculine *der* (mean_neut_der_ = 9.4%, SD = 10.7) compared to combinations with feminine *die* (mean_neut_die_ = 3.6%, SD = 6.7; t_1_(29) = 5.723, *p* < 0.001; t_2_(39) = 4.394, *p* < 0.001).

##### Reaction Times

**Fig. 3:**
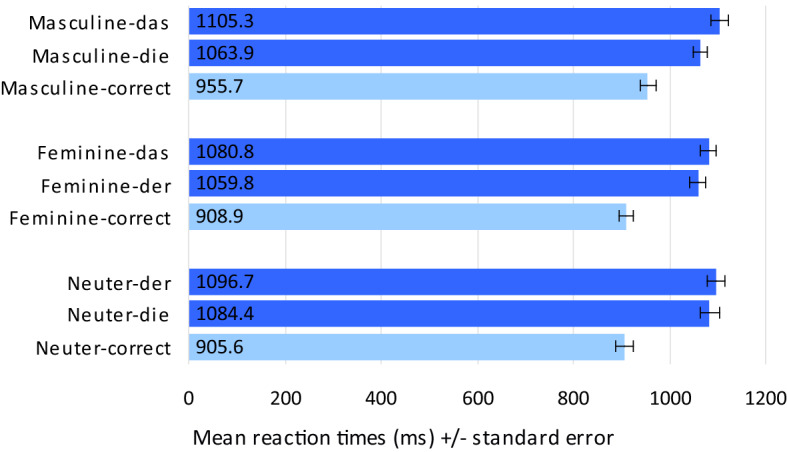
Mean reaction times (ms) in Experiment I (determiner noun congruence decision)

Results of the RT analyses are summarised in Fig. 3. Responses classified as errors (see above) were excluded from these analyses.

The RTs in the agreement violation conditions were compared with paired t-tests across participants and items.

*Agreement violation conditions for masculine nouns* Comparison of RTs in masculine nouns revealed a significant difference with longer RTs for combinations with neuter *das* (mean_mask_das_ = 1,105.3 ms, SD = 113.9) compared to combinations with feminine *die* (mean_mask_die_ = 1,063.9 ms, SD = 97.8; t_1_(29) = 3.581, *p* = 0.001; t_2_(38) = 3.208, *p* = 0.003).

*Agreement violation conditions for feminine nouns* Comparison of RTs in feminine nouns revealed a significant difference with longer RTs for combinations with neuter *das* (mean_fem_das_ = 1,080.8 ms, SD = 96.5) compared to combinations with masculine *der* (mean_fem_der_ = 1,059.8 ms, SD = 100.6; t_1_(29) = 2.195, *p* = 0.036; t_2_(39) = 2.406, *p* = 0.021).

*Agreement violation conditions for neuter nouns* Comparison of RTs in neuter nouns revealed no significant differences between combinations with masculine *der* (mean_neut_der_ = 1,096.7 ms, SD = 118.5) compared to combinations with feminine *die* (mean_neut_die_ = 1,084.4 ms, SD = 125.7; t_1_(29) = 0.938, *p* = 0.356; t_2_(39) = 1.379, *p* = 0.176).

#### Interim Discussion

In Experiment I, participants had to decide on gender agreement in visually presented determiner noun phrases. ERs and RTs were compared for the two agreement violation conditions of each target gender specification. It turned out that incongruent combinations with *das* resulted in higher ERs and RTs compared to incongruent combinations with *die* (and *der*). For neuter target nouns, incongruent combinations with *der* caused higher ERs but no RT differences compared to incongruent combinations with *die*. Different difficulties of detecting violations with one versus the other wrong determiner are unpredicted by categorial accounts, thus their explanation would need additional assumptions (e.g., based on frequency of use). While different difficulties of detecting violations with different determiners are, in principle, predicted by feature based accounts, the specific patterns observed did not fully agree with any of the predictions deduced from the previous studies.

However, it cannot be excluded that RTs in the present experiment were influenced by inherent characteristics of the determiners. For example, *der* [deːɐ̯] and *das* [das] consist of three phonemes each while *die* [diː] consists of only two. Furthermore, frequencies of the definite determiners differ. According to dlex (www.dlexdb.de), type frequency of *das* (absolute number of occurrences: 677,120) is lower than type frequency of *der* (3,026,098) and *die* (2,510,938).

For this reason, a second experiment was conducted, using the personal anaphoric pronouns *er* [eːɐ̯], *sie* [ziː], and *es* [ɛs] which all consist of two phonemes and are more similar regarding their type frequencies according to dlex (*er*: 604,723, *sie*: 607,179, *es*: 548,281).

### Experiment II: Gender Agreement Decision for Anaphoric Personal Pronouns and Nouns

Experiment II was a conceptual replication of Experiment I, differing in a) the function words used (personal anaphoric pronouns instead of definite determiners), b) the kind of stimulus presentation, and c) the participants.

#### Materials and Procedure

The procedure was analogous to Experiment I. This time, however, the anaphoric personal pronouns *e*r_masc_*, sie*_fem_*,* and *es*_neut_ were used as function words instead of definite determiners. Furthermore, presentation was not simultaneously in a left-to-right order. Instead, the pronoun was presented in the centre of the screen. After 600 ms, the noun appeared just below the pronoun. This was to accommodate for the fact that pronouns and nouns do not form immediate constituents of a single phrase in natural speech. Rather, they are connected by a paradigmatic relation. Presenting the pronoun first aimed at building up the expectation of a particular gender specification that could then be compared to the gender specification of the noun presented afterwards (instead of the other way round). Again, the participants had to decide on the agreement of gender specifications of pronoun and noun within a given pair.

As no compound filler stimuli had to be inserted in this experiment, the number of experimental stimuli could be increased with no extra effort for the participants. Thus, 180 morphologically simple nouns were used, 60 for each gender specification. One hundred and eighteen of them were taken from Experiment I (cf. Appendix Table 12 for a list of the stimuli). Each noun was presented with each of the three pronouns, thus calling for one YES answer (agreement) and two NO answers (agreement violations) per noun.

Testing was preceded by six practice trials in order to familiarise the participants with the task. The experiment consisted of 540 trials with pauses at an interval of 60 trials. Altogether, the experiment lasted approximately 30 min. RTs and correctness of the answers were recorded with DMDX.

#### Participants

Thirty-seven participants took part in Experiment II. None of them had taken part in Experiment I. Data of six participants had to be excluded from analysis due to bilingualism (*n* = 1), a technical error during data registration (*n* = 1), and ERs exceeding 10% (*n* = 4).^2^ Of the remaining 31 participants, 27 were female and four male. Their mean age was 22.6 years (range: 19–30 years). All participants included were native speakers of German. Two were left-handed. None of them was diagnosed with dyslexia. They were paid for their participation.

#### Results

##### Error Rates

**Fig. 4:**
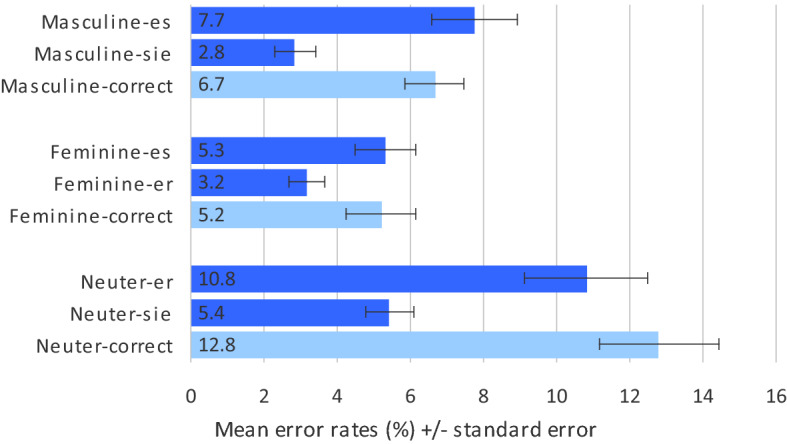
Mean error rates (%) in Experiment II (pronoun noun agreement decision)

Results of the ER analyses are summarised in Fig. 4. Agreement violation conditions are most relevant with respect to the different hypotheses.

The ERs in the agreement violation conditions were compared with paired t-tests across participants and items.

*Agreement violation conditions for masculine nouns* Comparison of ERs in masculine nouns revealed a significant difference with more errors on combinations with neuter *es* (mean_masc_es_ = 7.7%, SD = 9.1) compared to combinations with feminine *sie* (mean_masc_sie_ = 2.8%, SD = 4.4; t_1_(30) = 6.830, *p* < 0.001; t_2_(59) = 4.648, *p* < 0.001).

*Agreement violation conditions for feminine nouns* Comparison of ERs in feminine nouns revealed a significant difference with more errors on combinations with neuter *es* (mean_fem_es_ = 5.3%, SD = 6.3) compared to combinations with masculine *er* (mean_fem_er_ = 3.2%, SD = 3.73; t_1_(30) = 3.449, *p* = 0.002; t_2_(59) = 3.162, *p* = 0.002).

*Agreement violation conditions for neuter nouns* Comparison of ERs in neuter nouns revealed a significant difference with more errors on combinations with masculine *er* (mean_neut_er_ = 10.8%, SD = 13.0) compared to combinations with feminine *sie* (mean_neut_sie_ = 5.4%, SD = 5.2; t_1_(30) = 4.511, *p* < 0.001; t_2_(59) = 3.647, *p* = 0.001).

##### Reaction Times

**Fig. 5:**
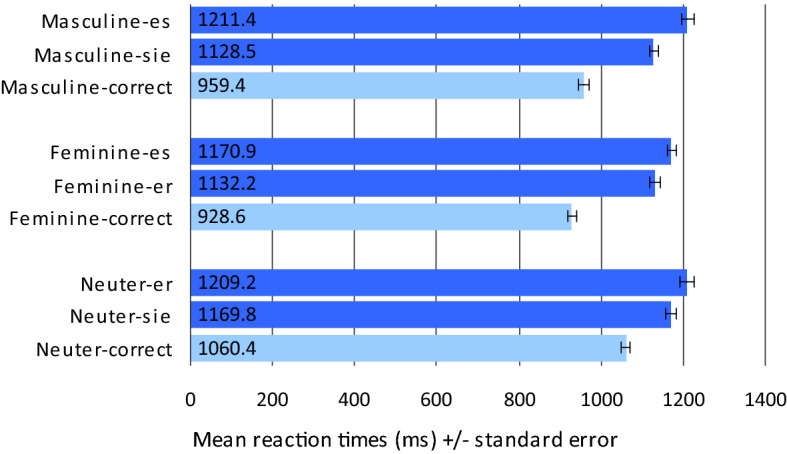
Mean reaction times (ms) in Experiment II (pronoun—noun agreement decision)

Results of the RT analyses are summarised in Fig. 5. Erroneous responses were excluded from these analyses. Again, agreement violation conditions are most relevant with respect to the different hypotheses.

The RTs in the agreement violation conditions were compared with paired t-tests across participants and items.

*Agreement violation conditions for masculine nouns* Comparison of RTs in masculine nouns revealed a significant difference with longer RTs for combinations with neuter *es* (mean_masc_es_ = 1,211.4 ms, SD = 108.5) compared to combinations with feminine *sie* (mean_masc_sie_ = 1,128.5 ms, SD = 94.2; t_1_(30) = 6.534, *p* < 0.001; t_2_(59) = 8.043, *p* < 0.001).

*Agreement violation conditions for feminine nouns* Comparison of RTs in feminine nouns revealed a significant difference with longer RTs for combinations with neuter *es* (mean_fem_es_ = 1,170.9 ms, SD = 79.9) compared to combinations with masculine *er* (mean_fem_er_ = 1,132.2 ms, SD = 95.8; t_1_(30) = 3.570, *p* = 0.001; t_2_(59) = 3.509, *p* = 0.001).

*Agreement violation conditions for neuter nouns* Comparison of RTs in neuter nouns revealed a significant difference with longer RTs for combinations with masculine *er* (MW_neut_er_ = 1.209,2 ms, SD = 118.4) compared to combinations with feminine *sie* (mean_neut_sie_ = 1,169.8 ms, SD = 99.4; t_1_(30) = 2.682, *p* = 0.012; t_2_(59) = 3.674, *p* = 0.001).

#### Interim Discussion

On the lines of Experiment I, in Experiment II errors and RTs were compared for gender agreement decisions on visually presented combinations of anaphoric personal pronouns and nouns. With only slight deviations, results of Experiment II equalled those of Experiment I and, again, differed from results of former studies. This was despite the fact that the anaphoric personal pronouns are more similar regarding phoneme number and frequency than the definite determiners.

Still, differences a) between individual processing times for the different determiners and pronouns and b) in acceptability of incorrect combinations of determiners or pronouns and nouns as described above might account for the diverging results. For this reason, regression analyses were conducted on the RTs of Experiments I and II including data from two control experiments.

### Regression Analyses on Results of Experiments I and II Including Data from Two Control Experiments

Before conducting regression analyses, two control experiments were run.

#### Control Experiment I: Lexical Decision for Definite Determiners and Personal Pronouns

As explicated above, determiners and pronouns differ regarding lexical features like grapheme and phoneme number, graphemic and phonological similarity, word frequency, frequency of appearance within inflectional paradigms, number of associated nouns and many others. Some of the relevant factors may even be still unknown. As it is impossible to control for all these factors, a lexical decision experiment was run in order to collect data on the processing of the determiners and pronouns used in Experiments I and II and, thus, obtain a measure of ‘processing costs’, reflecting the combined effect of relevant variables.

##### Materials

The definite determiners *der, die,* and *das* as well as the anaphorical pronouns *er, sie,* and *es* served as target stimuli in this control experiment. Nine more German function words served as fillers and 15 non-lexical two- or three-grapheme combinations served as nonwords (stimuli are listed in Appendix Table 13).

##### Procedure

Stimuli were displayed visually with the DMDX software in the centre of a computer screen. First, a fixation cross appeared for 600 ms. It was followed by the target item. Participants were instructed to decide as fast and accurately as possible about the lexical status of the target item by pressing the corresponding button (YES: word or NO: nonword). YES answers were assigned to the participant’s dominant hand. Every item was presented ten times, order of presentation being randomised. The testing was preceded by eight practice trials. Afterwards, all 300 experimental trials were run, with pauses at intervals of 100 trials. Overall, the control experiment took about ten minutes. Correctness of the answers and RTs were recorded with the DMDX software.

##### Participants

Thirty-five subjects participated in this control experiment. All of them had also participated in Experiment II. Thirty participants were female and five male. The mean age was 22.5 years (range: 19–30 years). All participants were native speakers of German. None of them was diagnosed with dyslexia. Two were left-handed. Participants were paid for their participation.

##### Results

Incorrect responses, responses lasting longer than 3,000 ms, and responses exceeding 2.5 standard deviations of a participant’s individual mean (calculated separately for YES and NO answers) were counted as errors. Across the 300 experimental stimuli, no participant exceeded an ER of 7% (mean ER: 3.8%, range: 2.0–7.0%). Descriptive statistical data for target determiners and pronouns are summarised in Table 4. Lexical decision times gathered in Control Experiment I were later used for the correction of RTs in regression analyses on the results of the main experiments.

**Table 4:** Mean lexical decision times (ms) for target definite determiners and anaphoric personal pronouns in Control Experiment I

der		die		das		er		sie		es	
Mean	SD	Mean	SD	Mean	SD	Mean	SD	Mean	SD	Mean	SD
575.9	19.9	551.9	15.1	559.6	17.9	585.0	19.2	550.5	11.2	584.1	19.7

#### Control Experiment II: Semantic and Formal Ratings of Noun Gender

In German, nouns differ regarding their semantic and formal characteristics which influence the probability of a particular gender assignment. For example, *Mann* „man“ is masculine due to biological sex of the referent. 90.4% of the nouns ending with schwa are feminine (Wegener, 1995, p. 76). All nouns with the suffix *–chen* are neuter. In some cases, there is a discrepancy of semantic and formal characteristics of a word (e.g., *das Mädchen*_neut_ “*the girl*”—the formal cue determines gender irrespective of the referent’s biological sex). As semantic and formal characteristics might result in different *perceived* degrees of gender (dis)agreement between a given determiner or pronoun and a given noun, a control experiment was run collecting data to explore the presence and metalinguistic awareness of such characteristics.

##### Materials

The same nouns were used as in Experiment II (thus also including all 118 stimuli used in Experiment I).

##### Procedure

Written target words were presented in randomised order one below the other in a column of a table. On the right, there were three empty columns. They were labelled as *masculine–feminine–neuter*, and coloured light blue, red, and green, respectively. Participants were instructed to rate on a scale of 1–10 as to how “masculine”, “feminine”, and “neuter” they perceived each noun. There was a semantic condition, in which participants were asked to concentrate on the meaning of the words, and there was a formal condition, in which they were asked to concentrate on the words’ form or sound when making their decision. Each noun was to be provided with three values (one for each gender specification). Participants were instructed that the individual values for each gender specification were independent from each other, i.e., they did not have to sum up to ten.

Semantic and formal ratings had to be carried out by the same participants but separately from each other. Half of the participants did the semantic ratings first, the other half started with the formal ratings. Word order was different in both conditions. Each rating run was preceded by six practice items. Overall, the investigation took 30–60 min.

##### Participants

Thirty-four participants took part in Control Experiment II. All of them had also participated in Experiment II. One subject had to be excluded from analysis due to incomplete completion of the form. Of the remaining 33 participants, 29 were female and four male. Their mean age was 22.3 years (range: 19–30 years).

##### Results

Altogether, two answers in the formal condition were missing. Thus, 17,820 semantic and 17,818 formal values were collected. Descriptive statistical data for the ratings of formal and semantic gender features of masculine, feminine, and neuter nouns are summarised in Table 5.

**Table 5:** Mean values of semantic and formal gender ratings of masculine, feminine, and neuter nouns (scale: 0–10) in Control Experiment II

	Semantic rating						Formal rating					
	masculine		feminine		neuter		masculine		feminine		neuter	
	Mean	SD	Mean	SD	Mean	SD	Mean	SD	Mean	SD	Mean	SD
Masculine nouns	**5.5**	**2.3**	2.6	1.3	5.4	2.1	**5.9**	**1.0**	2.9	1.1	4.9	0.8
Feminine nouns	2.2	1.1	**4.9**	**2.4**	5.6	2.1	2.8	1.0	**6.1**	**1.2**	4.6	0.8
Neuter nouns	3.5	1.4	2.9	1.4	**7.1**	**1.1**	3.8	1.2	3.3	1.3	**6.6**	**0.6**

Ratings corresponding to a noun’s gender type are given in bold

Based on the mean values of all participants, a semantic and a formal quotient were calculated for each noun, respectively, dividing the mean gender value corresponding to the correct gender specification of a given noun by the sum of the two gender values not corresponding to its gender specification. Thus, for example, the semantic values for *Hibiskus*_masc_ “*hibiscus*” were masculine = 2.00, feminine = 4.76, neuter = 6.12. This resulted in a semantic quotient for *Hibiskus* of 2.00: (4.76 + 6.12) = 0.18.

Semantic and formal quotients are supposed to indicate how much semantic and formal cues to gender are coded in a word, potentially resulting in an easier or more difficult decision on gender agreement. As can be seen in Table 5, the (correct) target gender usually yielded the highest ratings, both semantically and formally, but did not approach ceiling. In semantic ratings, feminine nouns had the highest feminine rating of the three noun types, but their own highest rating was neuter. In masculine nouns, masculine and neuter semantic ratings had similar values. This might be due to the fact that many words have no clear association with natural sex but are just ‘neuter’. Apart from that, alternative (incorrect) genders yielded lower ratings still substantially above bottom. Semantic and formal quotients were later included as predictor variables in regression analyses on the results of the main experiments.

#### Regression Analyses

Taking into consideration the results of the two control experiments, linear mixed effects regression analyses were conducted on RTs of correct trials in Experiments I and II (including the congruent as well as the incongruent determiner-noun phrases and pronoun-noun phrases) using the lme4 package (Bates et al., 2015) in R (R Core Team, 2018). Lexical decision times from Control Experiment I were subtracted from RTs in Experiments I and II in order to accommodate for the fact that determiners and pronouns differ regarding a set of factors which influence processing but cannot be fully controlled for. Corrected RTs, thus, are thought to reflect the time needed for processing of gender specification (and other higher-level representations) devoid of word processing costs. They served as dependent variable.

Most relevant potential predictor variables were Gender type of the noun (masculine / feminine / neuter) and Determiner (*der / die / das*) or Pronoun (*er / sie / es*), respectively. Other potential predictor variables included as fixed factors comprised Word length (number of graphemes), Word frequency (log10 lemma frequency according to dlex), Semantic quotient and Formal quotient (both yielded from Control Experiment II), Repetition (first, second, or third presentation of a given noun within the experiment), Position (consecutive number of a given item within the experiment), and Interaction of Frequency and Repetition. Participant was included as random factor.

##### Overall Analysis on Determiner-Noun Phrases in Experiment I

Results of the overall analysis on determiner-noun phrases (Experiment I) are summarised in Table 6. All predictor variables except for Semantic quotient influenced RTs. Specifically, RTs were faster for more frequent and shorter nouns and nouns with higher formal quotients. They decreased with increasing number of repetitions of a given noun and later positions within the experiment. Moreover, the influence of word frequency decreased with increasing number of repetitions of a given noun within the experiment.

**Table 6:** Overall regression analysis of corrected RTs in Experiment I

	Estimate	Std. Error	df	t-value	Pr( >|t|)	*p*
(Intercept)	701.50	36.98	78.62	18.97	4.52–31	< 0.001
Gender: feminine	− 159.54	9.24	9820.02	− 17.26	8.82–66	< 0.001
Gender: neuter	29.07	9.44	9820.01	3.08	2.08–03	0.002
Determiner: das	27.35	9.47	9820.05	2.89	3.89–03	0.004
Determiner: der	− 128.71	9.42	9820.03	− 13.67	3.86–42	< 0.001
Lemma frequency_log_	− 74.91	6.38	9820.02	− 11.74	1.34–31	< 0.001
Graphemes	16.27	1.24	9820.02	13.12	5.38–39	< 0.001
Formal quotient	− 30.17	9.72	9820.03	− 3.10	1.92–03	0.002
Semantic quotient	0.15	1.00	9820.01	0.15	8.82–01	0.882
Repetition	− 60.71	7.65	9820.04	− 7.93	2.35–15	< 0.001
Position	− 0.13	0.02	9820.26	− 6.53	6.80–11	< 0.001
Lemma frequency_log_*Repetition	13.36	2.77	9820.02	4.83	1.4–06	< 0.001
Feminine*das	138.55	13.06	9820.03	10.61	3.71–26	< 0.001
Neuter*das	− 212.95	13.21	9820.03	− 16.12	1.01–57	< 0.001
Feminine*der	255.74	13.02	9820.02	19.64	3.15–84	< 0.001
Neuter*der	121.31	13.20	9820.03	9.19	4.78–20	< 0.001

Overall, feminine nouns were processed faster than masculine nouns, and masculine nouns faster than neuter nouns. Combinations with *der* were processed faster than combinations with *die*, and combinations with *die* faster than combinations with *das*.

##### Analysis of Agreement-Violation Conditions in Experiment I

Additionally, pairwise comparisons among levels of factors were conducted with the emmeans-function in R (Lenth, 2022; cf. Table 7). They yielded faster RTs for congruent compared to incongruent combinations for each gender. Crucially, in the agreement-violation conditions, combinations with *die* and *der* were processed faster than combinations with *das* with masculine and feminine nouns, respectively. There was no significant difference in the RTs for combinations of *die* and *der* with neuter nouns.

**Table 7:** Pairwise comparisons of corrected RTs in Experiment I

Gender	Contrast	Estimate	SE	df	z.ratio	*p*
Masculine	die–das	− 27.4	9.47	Inf	− 2.887	0.011
	die–der	128.7	9.42	Inf	13.667	< 0.001
	das–der	156.1	9.57	Inf	16.305	< 0.001
Feminine	die–das	− 165.9	8.99	Inf	− 18.460	< 0.001
	die–der	− 127.0	9.00	Inf	− 14.119	< 0.001
	das–der	38.9	8.99	Inf	4.326	< 0.001
Neuter	die–das	185.6	9.20	Inf	20.169	< 0.001
	die–der	7.4	9.25	Inf	0.800	0.703
	das–der	− 178.2	9.34	Inf	− 19.069	< 0.001

##### Overall Analysis on Pronoun-Noun Phrases in Experiment II

Results of the overall analysis on pronoun-noun phrases (Experiment II) are summarised in Table 8. All predictor variables significantly influenced RTs. Specifically, RTs were faster for more frequent and shorter nouns and nouns with higher formal and semantic quotients. They decreased with increasing number of repetitions of a given noun and later positions within the experiment. Moreover, the influence of word frequency decreased with increasing number of repetitions of a given noun within the experiment.

**Table 8:** Overall regression analysis of corrected RTs in Experiment II

	Estimate	Std. Error	df	t-value	Pr( >|t|)	*p*
(Intercept)	910.95	44.24	58.96	20.59	1.31–28	< 0.001
Gender: feminine	− 193.06	8.97	15593.01	− 21.53	2.50–101	< 0.001
Gender: neuter	50.17	9.03	15593.01	5.56	2.79–08	< 0.001
Pronoun: es	43.88	8.98	15593.01	4.89	1.07–06	< 0.001
Pronoun: er	− 209.06	8.95	15593.01	− 23.37	1.03–118	< 0.001
Lemma frequency_log_	− 55.85	6.40	15593.02	− 8.72	2.96–18	< 0.001
Graphemes	12.11	1.32	15593.01	9.21	3.74–20	< 0.001
Formal quotient	− 59.03	7.87	15593.01	− 7.50	6.63–14	< 0.001
Semantic quotient	− 5.00	1.06	15593.01	− 4.72	2.37–06	< 0.001
Repetition	− 67.62	8.15	15593.01	− 8.29	1.19–16	< 0.001
Position	− 0.47	0.02	15593.02	− 24.68	6.27–132	< 0.001
Lemma frequency_log_*Repetition	11.12	2.80	15593.01	3.97	7.16–05	< 0.001
Feminine*es	168.63	12.69	15593.01	13.29	4.49–40	< 0.001
Neuter*es	− 198.38	12.83	15593.02	− 15.47	1.45–53	< 0.001
Feminine*er	378.04	12.63	15593.01	29.92	2.42–191	< 0.001
Neuter*er	207.02	12.77	15593.02	16.21	1.26–58	< 0.001

Again, overall feminine nouns were processed faster than masculine nouns, and masculine nouns faster than neuter nouns. Combinations with *er* were processed faster than combinations with *sie*, and combinations with *sie* faster than combinations with *es*.

##### Analysis of Agreement-Violation Conditions in Experiment II

As in Experiment I, pairwise comparisons (cf. Table 9) yielded faster RTs for congruent compared to incongruent combinations for each gender. Crucially, in the incongruent conditions, combinations with *sie* and *er* were processed faster than combinations with *es* with masculine and feminine nouns, respectively. There was no significant difference in the RTs for combinations of *sie* and *er* with neuter nouns.

**Table 9:** Pairwise comparisons of corrected RTs in Experiment II

Gender	Contrast	Estimate	SE	df	z.ratio	*p*
Masculine	sie–es	− 43.88	8.98	Inf	− 4.888	< 0.001
	sie–er	209.06	8.95	Inf	23.366	< 0.001
	es–er	252.94	9.06	Inf	27.911	< 0.001
Feminine	sie–es	− 212.51	8.97	Inf	− 23.692	< 0.001
	sie–er	− 168.97	8.92	Inf	− 18.946	< 0.001
	es–er	43.53	8.92	Inf	4.880	< 0.001
Neuter	sie–es	154.51	9.17	Inf	16.856	< 0.001
	sie–er	2.05	9.11	Inf	0.224	0.973
	es–er	− 152.46	9.30	Inf	− 16.397	< 0.001

##### Summary of Results

Overall, as in the studies of Opitz and colleagues, combinations with feminine nouns produced lowest reaction times. However, the observation of increased processing effort for masculine nouns (cf. Opitz & Pechmann, 2016; Opitz et al., 2013) was not confirmed. Congruent conditions were processed faster than incongruent conditions in both experiments. Incongruent combinations with *der* and *er* were processed faster than incongruent combinations with *die* and *sie*; incongruent combinations with *das* and *es* resulted in longest RTs.

As explicated above, we are cautious in interpreting the comparison of phrases containing nouns of different gender types as it seems virtually impossible to perfectly parallel these different nouns. Furthermore, the comparison of congruent vs. incongruent trials might reflect different processes involved in one but not the other (e.g., some kind of memorized visual picture which is recognized in the correct but not in the incorrect condition, e.g., Deutsch & Bentin, 2001). Therefore, the discussion focusses on the comparisons of the agreement violation conditions within each gender type, thus avoiding (a) the comparison of nouns of different gender types, and (b) possible different processing strategies related to the processing of congruent vs. incongruent phrases.

Results of the pairwise comparisons of RTs in the agreement violation conditions and of the t-tests conducted before are summarised in Table 10.

**Table 10:** Summary of results of RTs in the agreement violation conditions in Experiments I and II

	Masculine nouns	Feminine nouns	Neuter nouns
Experiment I: article-noun gender agreement			
T-tests on ERs	die < das**	der = das	der > die**
T-tests on RTs	die < das**	der < das*	der = die
Regression analyses on corrected RTs	die < das*	der < das**	der = die
Experiment II: pronoun-noun gender agreement			
T-tests on ERs	sie < es**	er < es**	er > sie**
T-tests on RTs	sie < es**	er < es**	er > sie*
Regression analyses on corrected RTs	sie < es**	er < es**	er = sie

*p < 0.01, **p < 0.001

## General Discussion

The present study aimed at testing hypotheses on the representation of grammatical gender within the mental lexicon. While prevailing psycholinguistic models of language processing assume categorial gender representation with one separate node for each gender specification (e.g., the traditional discrete two step model), more recently some authors have argued that mental representation and processing of grammatical gender parallels accounts of decomposition and underspecification put forward in theoretical linguistics. That is, gender specification in the mental lexicon may be based on feature representations instead of categorial gender nodes.

Against this background, two experiments were conducted with German speakers who had to decide on gender (dis-)agreement for visually presented combinations of I) definite determiners and nouns and II) anaphoric personal pronouns and nouns in an implicit nominative singular experimental setting. Each noun was combined with each determiner or pronoun, resulting in one agreement condition and two agreement violation conditions per noun.

Overall analyses showed fastest RTs for feminine nouns but no processing disadvantage for masculine nouns (or masculine determiners / pronouns) as predicted by Opitz et al. (2013) and Opitz and Pechmann (2016). Congruent trials were processed faster than incongruent trials. However, as nouns of the three gender types were not fully parallelised (and cannot be perfectly parallelised, after all) and correct vs. incorrect trials might evoke different cognitive processes, we focussed on the comparison of ERs and RTs in the agreement violation conditions separately per gender specification. Thus, the same nouns were compared within the same condition (gender disagreement) but with different incongruent determiners and pronouns, respectively. Overall, violations with neuter *das* / *es* yielded more processing effort than combinations with *die / sie* or *der / er*, while no general difference was found for combinations with *der / er* compared to *die / sie*.

### Categorial Versus Feature-Based Mental Representation of Gender

Differences between the two agreement violation conditions per gender specification as found in the present experiments clearly pose a challenge to models assuming categorial gender representation because three (in German) equivalent gender nodes should result in similar RTs or ERs irrespective of the type of agreement violation. Two objections can be raised, however.

First, language specific frequency differences between the gender specifications or the corresponding determiners and pronouns might account for (part of) the results. In fact, according to different counts of type and token frequency (e.g., Baayen et al., 2003; Wegera, 1997; Hoberg, 1999), neuter words are less frequent than masculine and feminine words in German. In consequence, as explicated above, neuter *das* is less frequent than *der* and *die*. However, a purely frequency-based explanation of our results is contradicted by the fact that the neuter pronoun *es* is not less frequent than *er* and *sie*. Nonetheless, *es* produced longer RTs in agreement violation conditions compared to *er* and *sie* in Experiment II. It is, thus, argued that frequency differences alone do not account for the observed behavioural differences between gender specifications.

Second, nouns differ regarding semantic and formal characteristics, which may serve as cues to their gender and might, in consequence, make one of the competing wrong gender specifications more probable than the other. For example, for German one-syllable nouns, Köpcke (1982) has described 24 phonological regularities for gender specification. Eleven of these rules only exclude one gender specification, but do not allow differentiating between the other two. According to Köpcke’s regularities, mostly, no differentiation between masculine and neuter is possible. This may be associated with more formal similarity between masculine and neuter nouns as compared to feminine nouns. The present study is the first one to meet such objections by taking individual semantic and formal quotients into consideration which are supposed to capture noun-inherent semantic and formal characteristics associated with one or the other gender specification. Indeed, these quotients did significantly influence RTs, but still left additional RT differences between different gender violation conditions. Thus, the results speak in favour of representations of grammatical gender in the mental lexicon that are more complex than categorial gender representation.

### Specific Accounts for Feature-Based Gender Representation and Processing

So far, three specific suggestions have been made regarding feature-based representation and processing of gender information.

According to Penke et al. (2004), dependent words are underspecified regarding gender with only positive features being part of the representation and neuter being the unmarked gender (masc [+ m], fem [+ f], neut []). Processing costs are supposed to result from the deviation of positive features of the dependent words that are missing in the context.

Opitz and colleagues (Opitz & Pechmann, 2016; Opitz et al., 2013) suggest a different kind of underspecification of the dependent words with feminine instead of neuter being the default gender (masc [+ m, − f], fem [], neut [− f]). While Opitz et al. (2013) interpret processing costs in terms of the differentiation of violation of compatibility vs. specificity, according to Opitz and Pechmann (2016) processing effort is directly related to the number of gender features involved.

These different suggestions lead to different predictions regarding error rates and reactions times in the experiments in this study (cf. Table 3). The results obtained contradict the predictions resulting from the accounts put forward by Opitz and colleagues. No evidence was found for the kind of underspecification they suggested. Instead, the results are consistent with the account of Penke et al. (2004).

On the basis of the present study, we consider the specific accounts of Opitz and colleagues as improbable. It does not follow, however, that the account of Penke and colleagues is the only possible explanation. As has been explicated in the introduction, their own study is not without limitations. Furthermore, feature-based processing comprises several variables like the type of underspecification, the specific kind of computation of processing costs, but also the question of context setting. A multitude of combinations of different specifications of these variables are possible, and the combination suggested by Penke et al. (2004) might only be one of those consistent with the results of our study. Furthermore, it has to be noted that our experimental setting represents a considerable simplification compared to natural language contexts, as only two-word phrases were presented, and a nominative singular context was implicitly induced. In natural language contexts, case and number add to the number and kind of features involved, and the linguistic contexts might be more or less explicit regarding their specification. Additionally, the situation is expected to be different in language systems other than German, which, for example, might contain only two or more than three gender types. So, the experimental paradigm used in the present study can only be a first step towards a comprehensive picture of gender representations in the mental lexicon.

## Conclusions

Altogether, the results of the experiments presented here call into question the assumption of categorial gender representation in the mental lexicon. Instead, they support the notion of feature-based mental representation and gender agreement processing. Future experiments will have to further support the specific explanation account of Penke et al. (2004) or to bring up another specific account into discussion and broaden the experimental setting in order to include further aspects of natural language processing.

## Data Availability

Data generated during and/or analysed during the current study are available from the corresponding author on reasonable request.

## Appendix

See Tables 11, 12 and 13.

**Table 11:** Stimuli included in Experiment I *Note:* Type frequencies and Lemma frequencies are taken from dlex

Target word	Translation	Gender	Graphemes	Phonemes	Syllables	Type frequency	Lemma frequency
Hibiskus	Hibiscus	m	8	8	3	7	7
Mozzarella	Mozzarella	m	10	8	4	5	5
Flamenco	Flamenco	m	8	8	3	9	11
Gorgonzola	Gorgonzola	m	10	10	4	13	13
Tollpatsch	Schlemiel	m	10	6	2	13	13
Mokassin	Moccasin	m	8	7	3	1	13
Sombrero	Sombrero	m	8	8	3	11	25
Wirsing	Savoy	m	7	6	2	27	48
Flamingo	Flamingo	m	8	8	3	13	45
Pinguin	Penguin	m	7	7	3	21	83
Astronaut	Astronaut	m	9	8	3	14	112
Parmesan	Parmesan	m	8	8	3	21	21
Bungalow	Bungalow	m	8	7	3	47	82
Pelikan	Pelican	m	7	7	3	32	86
Bumerang	Boomerang	m	8	7	3	34	36
Scharlatan	Charlatan	m	10	8	3	40	84
Alligator	Alligator	m	9	7	4	18	34
Schmetterling	Butterfly	m	13	8	3	317	899
Rosmarin	Rosemary	m	8	8	3	19	79
Baldrian	Valerian	m	8	8	3	21	37
Hengst	Stallion	m	6	5	1	49	342
Cousin	Cousin	m	6	5	2	118	146
Sohn	Son	m	4	3	1	13,477	18,899
Graf	Earl	m	4	4	1	1577	12,918
Prinz	Prince	m	5	5	1	270	5395
Mönch	Monk	m	5	4	1	694	1723
Ochse	Ox	m	5	4	2	145	699
Papst	Pope	m	5	5	1	3171	4427
Rüde	Male dog	m	4	4	2	6	46
Opa	Grandad	m	3	3	2	441	484
Hai	Shark	m	3	2	1	82	247
Efeu	Ivy	m	4	3	2	161	170
Mund	Mouth	m	4	4	1	10,108	12,949
Kopf	Head	m	4	3	1	27,358	33,309
Bauch	Stomache	m	5	3	1	2376	2759
Fisch	Fish	m	5	3	1	1988	5218
Roggen	Rye	m	6	4	2	602	636
Hals	Throat	m	4	4	1	4698	5106
Ingwer	Ginger	m	6	4	2	54	75
Affe	Monkey	m	4	3	2	363	1268
Parodontose	Periodontosis	f	11	11	5	1	2
Gastronomie	Gastronomy	f	11	10	4	39	39
Pistazie	Pistachio	f	8	8	3	4	25
Lakritze	Liquorice	f	8	7	3	10	17
Frikadelle	Meatball	f	10	9	4	3	27
Schnulze	Sob stuff	f	8	6	2	13	21
Zwetschge	Plum	f	9	7	2	9	34
Hagebutte	Rose hip	f	9	8	4	11	67
Schramme	Scratch	f	8	5	2	58	139
Schleuder	Catapult	f	9	5	2	60	175
Harpune	Harpoon	f	7	7	3	36	55
Schatulle	Jewel case	f	9	6	3	76	96
Aubergine	Aubergine	f	9	8	4	9	32
Pinzette	Tweezers	f	8	7	3	77	96
Remoulade	Remoulade	f	9	8	4	20	20
Konfitüre	Jam	f	9	9	4	39	62
Plakette	Badge	f	8	7	3	50	95
Tombola	Tombola	f	7	7	3	69	71
Hornisse	Hornet	f	8	7	3	34	95
Kartusche	Cartridge	f	9	7	3	18	55
Stute	Female horse	f	5	5	2	227	342
Sau	Sow	f	3	2	1	322	406
Oma	Grandma	f	3	3	2	570	621
Nonne	Nun	f	5	4	2	295	696
Hexe	Witch	f	4	5	2	630	953
Henne	Hen	f	5	4	2	204	351
Tante	Aunt	f	5	5	2	4703	4997
Schwester	Sister	f	9	6	2	6391	8272
Nixe	Mermaid	f	4	5	2	33	86
Fee	Fairy	f	3	2	1	137	226
Wespe	Wasp	f	5	5	2	77	242
Maus	Mouse	f	4	3	1	754	1293
Bohne	Bean	f	5	4	2	161	767
Beere	Berry	f	5	4	2	48	420
Rose	Rose	f	4	4	2	6	2514
Eiche	Oak	f	5	3	2	375	377
Blume	Flower	f	5	5	2	1269	5446
Nase	Nose	f	4	4	2	5454	5852
Distel	Thistle	f	6	5	2	18	131
Milz	Spleen	f	4	4	1	178	178
Stethoskop	Stethoscope	n	10	9	3	38	39
Frikassee	Fricassee	n	9	7	3	27	28
Rhinozeros	Rhinoceros	n	10	9	4	20	20
Trampolin	Trampoline	n	9	8	3	7	12
Perlmutt	Mother of pearl	n	8	7	2	27	28
Projektil	Projectile	n	9	9	3	14	40
Skalpell	Scalpel	n	8	7	2	39	45
Känguru	Kangaroo	n	8	7	3	35	64
Chamäleon	Chameleon	n	9	8	4	58	84
Scharnier	Hinge	n	9	6	2	41	106
Akkordeon	Accordion	n	9	8	4	72	78
Tandem	Tandem	n	6	6	2	28	49
Karussell	Carousel	n	9	7	3	160	200
Dromedar	Dromedary	n	8	8	3	23	32
Konzentrat	Concentrate	n	10	10	3	23	35
Planetarium	Planetarium	n	11	11	5	17	31
Impressum	Imprint	n	9	8	3	48	50
Resümee	Resume	n	7	6	3	78	82
Minarett	Minaret	n	8	7	3	48	89
Chlorophyll	Chlorophyll	n	11	8	2	82	100
Reh	Deer	n	3	2	1	178	368
Baby	Baby	n	4	4	1	978	1203
Ohr	Ear	n	3	2	1	3810	8102
Pferd	Horse	n	5	4	1	3572	9334
Kinn	Chin	n	4	3	1	1594	1687
Auge	Eye	n	4	3	2	9535	47,966
Lama	Lama	n	4	4	2	76	134
Herz	Heart	n	4	4	1	10,701	20,087
Fleisch	Meat	n	7	4	1	4927	5450
Kind	Child	n	4	4	1	20,924	60,369
Rad	Wheel	n	3	3	1	1275	2808
Auto	Car	n	4	3	2	4440	6342
Kanu	Canoe	n	4	4	2	28	66
Zelt	Tent	n	4	4	1	852	1529
Radio	Radio	n	5	5	3	3428	3534
Kleid	Dress	n	5	4	1	2262	5853
Moped	Moped	n	5	5	2	37	78
Kino	Cinema	n	4	4	2	1444	1850
Sieb	Strainer	n	4	3	1	287	339
Dorf	Village	n	4	4	1	4596	8305

**Table 12:** Stimuli included in Experiment II and Control Experiment II *Note:* Type frequencies and Lemma frequencies are taken from dlex

Target word	Translation	Gender	Graphemes	Phonemes	Syllables	Type frequency	Lemma frequency
Rat	Council	m	3	3	1	8194	11915
Knast	Clink	m	5	5	1	199	224
Saal	Hall	m	4	3	1	3521	4751
Lack	Varnish	m	4	3	1	352	540
Raps	Rape	m	4	4	1	143	150
Wodka	Vodka	m	5	5	2	300	305
Abend	Evening	m	5	4	2	13856	17447
Scheck	Cheque	m	6	3	1	367	614
Schutz	Protection	m	6	4	1	7389	8941
Acker	Field	m	5	3	2	743	1218
Teddy	Teddy	m	5	4	1	129	304
Gorilla	Gorilla	m	7	6	3	118	162
Mozzarella	Mozzarella	m	10	9	4	5	5
Gorgonzola	Gorgonzola	m	10	10	4	13	13
Sombrero	Sombrero	m	8	8	3	11	25
Flamenco	Flamenco	m	8	8	3	9	11
Flamingo	Flamingo	m	8	8	3	13	45
Tornado	Tornado	m	7	7	3	178	225
Kakadu	Cockatoo	m	6	6	3	30	50
Leopard	Leopard	m	7	7	3	84	208
Magen	Stomach	m	5	4	2	1943	2224
Mantel	Coat	m	6	5	2	3233	4172
Vogel	Bird	m	5	4	2	2359	5919
Muskel	Muscle	m	6	5	2	434	2093
Tollpatsch	Schlemiel	m	10	7	2	13	13
Mokassin	Moccasin	m	8	7	3	1	13
Wirsing	Savoy	m	7	6	2	27	48
Pinguin	Penguin	m	7	7	3	21	83
Astronaut	Astronaut	m	9	9	3	14	112
Parmesan	Parmesan	m	8	8	3	21	21
Bungalow	Bungalow	m	8	7	3	47	82
Pelikan	Pelican	m	7	7	3	32	86
Bumerang	Boomerang	m	8	7	3	34	36
Scharlatan	Charlatan	m	10	8	3	40	84
Alligator	Alligator	m	9	8	4	18	34
Schmetterling	Butterfly	m	13	8	3	317	899
Rosmarin	Rosemary	m	8	8	3	19	79
Baldrian	Valerian	m	8	8	3	21	37
Hibiskus	Hibiscus	m	8	8	3	7	7
Bogen	Bow	m	5	4	2	2556	2916
Faden	Thread	m	5	4	2	1294	2597
Hai	Shark	m	3	3	1	82	247
Papagei	Parrot	m	7	7	3	244	422
Mund	Mouth	m	4	4	1	10108	12949
Kopf	Head	m	4	4	1	27358	33309
Bauch	Stomache	m	5	4	1	2376	2759
Fisch	Fish	m	5	3	1	1988	5218
Roggen	Rye	m	6	4	2	602	636
Hals	Throat	m	4	4	1	4698	5106
Ingwer	Ginger	m	6	4	2	54	75
Hengst	Stallion	m	6	5	1	49	342
Cousin	Cousin	m	6	4	2	118	146
Sohn	Son	m	4	3	1	13477	18899
Graf	Earl	m	4	4	1	1577	12918
Prinz	Prince	m	5	6	1	270	5395
Mönch	Monk	m	5	4	1	694	1723
Ochse	Ox	m	5	4	2	145	699
Papst	Pope	m	5	5	1	3171	4427
Rüde	Male dog	m	4	4	2	6	46
Opa	Grandad	m	3	3	2	441	484
Tat	Act	f	3	3	1	10222	12431
Gruft	Vault	f	5	5	1	247	301
Bahn	Train	f	4	3	1	3832	5371
Naht	Seam	f	4	3	1	297	541
Milz	Spleen	f	4	4	1	178	178
Flora	Flora	f	5	5	2	408	430
Tugend	Virtue	f	6	5	2	1641	2577
Fracht	Load	f	6	5	1	282	388
Schuld	Guilt	f	6	4	1	5302	7078
Leber	Liver	f	5	4	2	873	930
Party	Party	f	5	5	1	924	961
Tombola	Tombola	f	7	7	3	69	71
Harmonika	Harmonica	f	9	9	4	89	94
Malaria	Malaria	f	7	7	4	255	257
Parodontose	Periodontosis	f	11	11	5	1	2
Gastronomie	Gastronomy	f	11	10	4	39	39
Pistazie	Pistachio	f	8	9	3	4	25
Lakritze	Liquorice	f	8	8	3	10	17
Villa	Villa	f	5	4	2	1595	2098
Firma	Company	f	5	5	2	4670	7372
Gala	Gala	f	4	4	2	32	59
Insel	Island	f	5	4	2	5098	7409
Regel	Rule	f	5	4	2	6881	10321
Wurzel	Root	f	6	5	2	1491	3634
Oper	Opera	f	4	3	2	5166	6591
Frikadelle	Meatball	f	10	9	4	3	27
Schnulze	Sob stuff	f	8	7	2	13	21
Zwetschge	Plum	f	9	8	2	9	34
Hagebutte	Rose hip	f	9	8	4	11	67
Schramme	Scratch	f	8	5	2	58	139
Schleuder	Catapult	f	9	5	2	60	175
Harpune	Harpoon	f	7	7	3	36	55
Schatulle	Jewel case	f	9	6	3	76	96
Aubergine	Aubergine	f	9	8	4	9	32
Pinzette	Tweezers	f	8	8	3	77	96
Remoulade	Remoulade	f	9	8	4	20	20
Konfitüre	Jam	f	9	9	4	39	62
Plakette	Badge	f	8	7	3	50	95
Hornisse	Hornet	f	8	7	3	34	95
Kartusche	Cartridge	f	9	7	3	18	55
Mauer	Wall	f	5	4	2	3335	5396
Wespe	Wasp	f	5	5	2	77	242
Maus	Mouse	f	4	4	1	754	1293
Bohne	Bean	f	5	4	2	161	767
Beere	Berry	f	5	4	2	48	420
Rose	Rose	f	4	4	2	6	2514
Eiche	Oak	f	5	4	2	375	377
Blume	Flower	f	5	5	2	1269	5446
Nase	Nose	f	4	4	2	5454	5852
Distel	Thistle	f	6	5	2	18	131
Stute	Female horse	f	5	5	2	227	342
Sau	Sow	f	3	3	1	322	406
Oma	Grandma	f	3	3	2	570	621
Nonne	Nun	f	5	4	2	295	696
Hexe	Witch	f	4	5	2	630	953
Henne	Hen	f	5	4	2	204	351
Tante	Aunt	f	5	5	2	4703	4997
Schwester	Sister	f	9	6	2	6391	8272
Nixe	Mermaid	f	4	5	2	33	86
Fee	Fairy	f	3	2	1	137	226
Amt	Department	n	3	3	1	6792	11470
Biest	Beast	n	5	4	1	169	228
Heer	Army	n	4	3	1	3226	6273
Joch	Saddle	n	4	3	1	434	602
Malz	Malt	n	4	5	1	103	124
Komma	Comma	n	5	4	2	266	313
Elend	Misery	n	5	5	2	2131	2425
Schilf	Reed	n	6	4	1	301	435
Schiff	Ship	n	6	3	1	4565	10744
Leder	Leather	n	5	4	2	1085	1122
Hobby	Hobby	n	5	4	2	135	184
Aroma	Flavour	n	5	5	3	220	260
Paradigma	Paradigm	n	9	9	4	182	263
Panorama	Panorama	n	8	8	4	215	276
Allegro	Allegro	n	7	6	3	124	145
Inferno	Inferno	n	7	7	3	100	133
Embargo	Embargo	n	7	7	3	95	138
Fiasko	Fiasco	n	6	6	3	155	163
Klima	Climate	n	5	5	2	1449	1713
Thema	Topic	n	5	4	2	6829	9713
Zebra	Zebra	n	5	6	2	22	73
Stethoskop	Stethoscope	n	10	9	3	38	39
Frikassee	Fricassee	n	9	7	3	27	28
Rhinozeros	Rhinoceros	n	10	10	4	20	20
Trampolin	Trampoline	n	9	9	3	7	12
Perlmutt	Mother of pearl	n	8	7	2	27	28
Projektil	Projectile	n	9	9	3	14	40
Skalpell	Scalpel	n	8	7	2	39	45
Känguru	Kangaroo	n	7	7	3	35	64
Chamäleon	Chameleon	n	9	8	4	58	84
Scharnier	Hinge	n	9	6	2	41	106
Akkordeon	Accordion	n	9	8	4	72	78
Tandem	Tandem	n	6	6	2	28	49
Karussell	Carousel	n	9	7	3	160	200
Dromedar	Dromedary	n	8	8	3	23	32
Konzentrat	Concentrate	n	10	11	3	23	35
Planetarium	Planetarium	n	11	11	5	17	31
Impressum	Imprint	n	9	8	3	48	50
Resümee	Resume	n	7	6	3	78	82
Minarett	Minaret	n	8	7	3	48	89
Chlorophyll	Chlorophyll	n	11	8	2	82	100
Baby	Baby	n	4	4	1	978	1203
Auge	Eye	n	4	4	2	9535	47966
Lama	Lama	n	4	4	2	76	134
Zelt	Tent	n	5	5	1	852	1529
Radio	Radio	n	5	5	3	3428	3534
Kleid	Dress	n	5	5	1	2262	5853
Auto	Car	n	4	4	2	4440	6342
Kino	Cinema	n	4	4	2	1444	1850
Sieb	Strainer	n	4	3	1	287	339
Reh	Deer	n	3	2	1	178	368
Ohr	Ear	n	3	2	1	3810	8102
Pferd	Horse	n	5	5	1	3572	9334
Kinn	Chin	n	4	3	1	1594	1687
Herz	Heart	n	4	5	1	10701	20087
Fleisch	Meat	n	7	5	1	4927	5450
Kind	Child	n	4	4	1	20924	60369
Rad	Wheel	n	3	3	1	1275	2808
Kanu	Canoe	n	4	4	2	28	66
Moped	Moped	n	5	5	2	37	78

**Table 13:** Stimuli included in Control Experiment I

Category	Target word	Translation
Determiner	der	the_masc_
Determiner	die	the_fem_
Determiner	das	the_neut_
Pronoun	er	he
Pronoun	sie	she
Pronoun	es	it
Filler	auf	on
Filler	bei	at
Filler	mit	with
Filler	wir	we
Filler	ihr	you
Filler	für	for
Filler	zu	to
Filler	an	on
Filler	so	so
Nonword	eul	–
Nonword	laf	–
Nonword	dun	–
Nonword	bes	–
Nonword	pir	–
Nonword	dob	–
Nonword	mat	–
Nonword	dög	–
Nonword	nif	–
Nonword	ilt	–
Nonword	os	–
Nonword	un	–
Nonword	bü	–
Nonword	fo	–
Nonword	eb	–

## Footnotes

1 Dlex (www.dlexdb.de) is an online lexical database which provides norms of word properties for psychological and linguistic research. It is based on the *Kernkorpus des Digitalen Wörterbuchs der deutschen Sprache* (DWDS) with over 100 million running words (cf. Heister et al., 2011).

2 Incorrect reactions, reactions lasting longer than 3,000 ms, and reactions exceeding 2.5 standard deviations of a participant’s mean (calculated separately for YES and NO answers) were counted as errors.

## Code Availability

Data generated during and/or analysed during the current study are available from the corresponding author on reasonable request.
